# Evaluation of Verapamil Efficacy in Peyronie’S Disease Comparing With Pentoxifylline

**DOI:** 10.5539/gjhs.v6n7p23

**Published:** 2014-09-18

**Authors:** M. Alizadeh, F. Karimi, M. R. Fallah

**Affiliations:** 1 Nephrology and kidney Transplant Research Center, Urmia University of Medical Sciences, Urmia, Iran; 2 Urmia University of Medical Sciences, Urmia, Iran

**Keywords:** pentoxifylline, verapamil, Peyronie’s disease

## Abstract

**Introduction:**

Peyronie’s disease described as penile curvature, fibromathosis and pain that occur most often in men aged 40 to 60 years. The main complaint that caused the patient to visit the clinic is nodules on the upper surface of the penis, causing curvature and distortion particularly during erection, but they don’t have any urinary problem. In this study, we evaluated the effect of verapamil compared to pentoxifylline in Peyronie’s disease.

**Method:**

In this study, 90 patients with signs and symptoms of Peyronie’s disease which were diagnosed and were in the age range 40 to 70 years enrolled. The patients were randomly divided into 3 groups. First group received pentoxifylline orally at a dose of 400 mg three times a day, in the second group verapamil (10 mg every other week for up to 12 sessions) was injected into the lesion and the third group received both treatments in combination.

**Results:**

In patients, who received pentoxifylline, curvature reduction was 26.7%, plaque size reduction was 30%, the recovery rate of erectile dysfunction was 46.7% and pain reduced was 73.3%. Each of these cases in patients, who used beta-blockers, was 36.7%, 33.3%, 66.7% and 76.6%. In combination therapy, curvature reduction was 36.7%, plaque size reduction was 33.3%, the recovery rate of erectile dysfunction was 86.7% and pain reduced was 80%.

**Conclusion:**

In our study there was no significant difference between two groups using verapamil or pentoxifylline, but there was a significant improvement in combination therapy group. Due to our results we propose that combination therapy can improve results and should be considered as a choice in treatment of Peyronie’s disease.

## 1. Introduction

The famous French surgeon Dr. Lapeyronie described the first details about a disease from nodular diseases of cave bodies that cause deviation that it was named in his honor Peyronie’s disease (Iacono et al., 1993). The disease is almost synonymous with the kind of penile fibromatos disease affects one percent of white men and therefore considered a difficulty for the urologist and is considered a common disease (Bella et al., 2007). Peyronie’s disease with two definitions of fibromatos and penile curvature and erectile dysfunction mostly occur in men aged 40 to 60 (Gonzalez-Cadavid, 2009), but like any other disease in the area of empirical sciences exceptionally analyzed and reported in younger men (De Young & Bella, 2010). The main complaint that makes a visit to the clinic, is of creating fibromatos nodules on the upper surface of the penile, which leads to the curvature and deviation of penile especially during erection, while create no problem with urinary in terms of urine (Pohlers et al., 2009). The main problem created by the diversion is disruption during intercourse, pain during sexual intercourse and abnormality in rhythm of sexual movements (El-Sakka et al., 1997a). Fibromatos nodular disease and Peyronie’s disease is related to the connective tissue and elastic diseases and the overall course of the disease is divided into two stages. First phase is characterized by the onset of disease in inflammatory and active phase of the disease and with penile pain during intercourse without any diversion or obstruction disorder in the urinary tract (El-Sakka et al., 1997b; Moreland et al., 1995). The aetiology of this stage is associated with crescent-shaped superficial vein thrombosis and also deep veins that its diagnosis is known by a typical hypodense region in the septum or in the posterior tunica of penile (Cutroneo, 2007). This process takes about 9 to 15 months to reach the second stage in the natural history of the disease, including fibrosis stage (Kuang et al., 2007). In this stage the first phenomenon is the formation of connective tissue plaques. Establishing the nature of the fibrous tunica albuginea creates less elasticity and so distortion and pain during intercourse in the penile (Wagenseil & Mecham, 2007). Only a small number of drugs based on the specifications based on evidence have tested and two products one as oral (pentoxifylline) and the other as injection of nodules (verapamil) have shown relatively good therapeutic effect in the natural history of the disease (Stewart, 1994; Akkus et al., 1997). TGF-B1 has underlying role in the pathogenesis of Peyronie’s disease and elastin and collagen metabolism (Deree et al., 2007; Hung et al., 2008). Pentoxifylline, which is an antagonist of TGF-B, in doses of 100 micro moles per liter prevent the proliferation of fibroblasts (Lin, 2005). TGF-B1 will perform elastogen mobility and deposition of collagen I fibers in TADF’s (fibroblasts derived from tunica albuginea) in a given time and dose (Ng, 2009; Valente et al., 2003) Pretreatment with pentoxifylline decreases significantly elastogen by TGF-B1 and deposition of collagen fibers in TADF’s in people with and without Peyronie’s disease (Brant, Dean, & Lue, 2006; Lin, 2005). Collagen I synthesis in patients’ tunica with Peyronie who were not treated is more than the tunica of normal people. (Mulhall et al., 2005) And pentoxifylline may reduce collagen synthesis (Lin, 2004). This effect is seen in the tunica of patients with Peyronie’s disease and cannot be seen in the tunica of healthy people (Ten Dijke & Hill, 2004; Derynck & Zhang, 2003). This indicates the differences between normal cells tunica and tunica of patients with Peyronie’s disease (Hirai et al., 2007). Making elastin and collagen is stimulated by TGF-B1 in TADF’s that causes Peyronie’s disease phenotype formation (Hassoba, El-Sakka, & Lue, 2005). Pretreatment with pentoxifylline reduces deposition of collagen fibers and elastogen in TADF’s that are exposed to TGF-B1 that this effect is a useful role of pentoxifylline in the treatment of Peyronie’s disease (Bivalacqua et al., 2000; Mulhall et al., 2002). An intralesional injecting therapy involves a direct injection into the plaque. In this way to achieve a high local concentration by avoiding systemic side effects can be created. No medication should help solve or at least soften the plaque. Signs should either improve or disappear entirely but it should be noted that each injection is painful as well as we cannot rule out the damage resulting from the injection of an inflammatory process. It is believed that calcium channel blockers such as verapamil by calcium-dependent secretion of macromolecules from fibroblasts reduced collagen production and enhances collagenase activity, also inhibits the synthesis and secretion of fibronectin (Del Carlo, Cole, & Levine, 2008; Szardening-Kirchner et al., 2009). In a study conducted in a number of patients with Peyronie after receiving a dozen sessions of intralesional verapamil (every two weeks at a rate of ten milligrams) shows one hundred percent pain reduction and thirty percent plaque reduction and seventy-six percent penile deviation regression and seventy-two percent improvement in sexual activity of patients (Hoff, Perkins, & Davidson, 1999). In another study performed on patients with Peyronie which amounted to four hundred milligrams of pentoxifylline orally twice a day generally received thirty-seven percent of positive therapeutic response (Cantor, Keller, Mandl, & Turino, 1987). But, effective studies have not been conducted yet in connection to efficiency and effectiveness of these two drugs and their combination in the control of symptoms and stop or slow the disease process and improve symptoms. Hence, we investigated the effects of two injectable verapamil and oral pentoxifylline and the combination of these two drugs in the treatment of the amount of pain, the size of the plaque, the amount of circumflexion, erectile dysfunction and the control of progress of the disease and improvement of symptoms of peyronie’s disease. It also appears that receiving a combination of two pentoxifylline with mechanism of reduced collagen fiber deposition and elastogen and intralesional verapamil with mechanism of inhibition of synthesis and secretion of fibronectin and also decreasing collagen production and increasing the activity of collagenase had a greater improving effects to receive each drugs in the treatment protocol of Peyronie’s disease alone. At the end of the study and statistical survey, if possible, an appropriate treatment and suggestions to improve treatment process that is providing presently which focuses on the second stage of the course that has stronger and more serious malignant changes.

## 2. Materials and Methods

In this empirical study a total of 90 patients with signs and symptoms of the peyronie’s disease were enrolled in the age range 40 to 70 years. Excluding parameters are including patients that taken treatment, surgery, trauma, investing tool, radiotherapy, or underwent a treatment protocol as well as patients who have diabetes or taking alcohol or other significant underlying disease other than Peyronie were also excluded from the study. Then patients were randomly divided into 3 groups of 30 and one oral pentoxifylline group at the dose of 400 mg three times a day and the next group intralesional verapamil (10 m every other week for up to 12 sessions) injected and finally, the third group received both treatments in combination. Then the co-trainer, who was trained about Peyronie’s disease and the study, over the course of 6 months’ treatment protocol, plaque size and nature change, deviation or curvature of the penile, pain, level of erectile dysfunction and changes in sexual function of patients in both groups were asked and viewed and raw data was recorded. The recorded data were analyzed and the results were recorded. In our study, the recovery rate of variables (erectile dysfunction, size of curvature, plaque size and pain) was considered before and after intervention. Improvement in erectile dysfunction: if the rate of improvement in erectile dysfunction before and after the intervention based on IIEF was more than 10 score was considered as improvement in erectile dysfunction, and if it was lower was considered as a failure to improve erectile dysfunction. Curve reduction: if the curve reduction rate before and after the intervention was greater than10 ° was considered as the curve reduction. Plaque size reduction: if reduction in the size of the plaque before and after the intervention was greater than 3 mm was considered as a reduction in plaque size. Pain reduction: if the pain reduction rate before and after the intervention was more than 2 score was considered as pain reduction. For data analysis of this study Spss 16 software was used (Majazi-Dalfard et al., 2013).

## 3. Results

In this quasi-experimental study, 90 patients with signs and symptoms of Peyronie’s disease diagnosis that is given to them in the age range 40 to 70 years were enrolled. According to the results of Table 1, 30 patients were administered pentoxifylline and the efficacy of these medicines was studied in patients and the results showed:

**Table 1:** Determine the efficacy of oral pentoxifylline therapy (plaque size, curvature size, erectile dysfunction and pain)

	Improvement (reduced effect)	
Variable	Have	Do not have
Curvature reduction	8(26.7%)	22(73.3%)
Plaque volume reduction	9(30%)	21(70%)
Improving erectile dysfunction	14(46.7%)	16(53.3%)
Pain rate reduction	22(73.3%)	8(26.7%)

1) 8 patients (26.7%) had curvature reduction and 22 patients (73.3%) were without reduction in curvature.
2) Plaque volume in 9 patients (30%) was decreased and in 21 patients (70%) was not decreased.
3) Erectile dysfunction in 14 patients (46.7%) improved and in 16 patients (53.3%) had no improvement.
4) Pain in 22 patients (73.3%) was decreased and in 8 patients (26.7%) no decrease in pain was reported.

Table 2 also shows the efficiency of the verapamil in 30 patients in this study were as follows: 11 patients (36.7%) decrease in curvature and 19 patients (63.3%) had not decrease the curvature. Plaque size in 10 patients (33.3%) was decreased and in 20 patients (66.7%) had not decreased. Erectile dysfunction in 20 patients (66.7%) was improved and in 10 patients (33.3%) had no improvement. Pain in 23 of 30 patients (76.7%) was decreased and in 7 patients (23.3%) did not report pain relief.

**Table 2:** Determine the efficacy of verapamil therapy (plaque size, curvature size, erectile dysfunction and pain)

	Improvement (reduced effect)	
Variable	Have	Do not have
Curvature reduction	11(36.7%)	19(63.3%)
Plaque volume reduction	10(33.3%)	20(66.7%)
Improving erectile dysfunction	20(66.7%)	10(33.3%)
Pain rate reduction	23(76.7%)	7(23.3%)

Verapamil with oral pentoxifylline was administered to 30 patients and efficacy of this drug in such patients was as the follows: 11 patients (36.7%) had decrease in curvature and 19 patients (63.3) had not decrease in the curvature. Plaque size in 10 patients (33.3%) was decreased and in 20 patients (66.7%) was not decreased. Erectile dysfunction in 26 patients (86.7%) was improved and in 4 patients (13.3%) had no improvement. Reducing pain in 30 patients receiving verapamil and oral pentoxifylline was as: in 24 patients (80%) the pain was reduced and in 6 patients (20%) did not reduce the amount of pain (Table 3).

**Table 3:** Determine the efficacy of verapamil and pentoxifylline therapy (plaque size, curvature size, erectile dysfunction and pain

	Improvement (reduced effect)	
Variable	Have	Do not have
Curvature reduction	11(36.7%)	19(63.3%)
Plaque volume reduction	10(33.3%)	20(66.7%)
Improving erectile dysfunction	26(86.7%)	4(13.3%)
Pain rate reduction	24(80%)	6(20%)

Given Table 4, comparison of efficiency of pentoxifylline with verapamil showed, recovery rate of curve in patients receiving pentoxifylline was (26.7%) and verapamil was (36.7%), respectively. According to fisher exact statistical analysis, there is no significant differences among the three groups receiving pentoxifylline with verapamil and pentoxifylline in curvature reduction (P=0.63). In this study verapamil with pentoxifylline and verapamil in terms of curve reduction are not significantly different (P=0.6) also there in no significant difference between pentoxifylline and pentoxifylline with verapamil (P=0.29)

**Table 4:** Comparison of the efficacy of Pentoxifylline - Verapamil - Pentoxifylline and Verapamil in curve reduction

Total	Curve reduction		Treatment with

	Do not have	Have	
30(100%)	22(73.3%)	8(26.7%)	Pentoxifylline
30(100%)	19(63.3%)	11(36.7%)	Verapamil
30(100%)	19(63.3%)	11(36.7%)	Pentoxifylline and Verapamil
90(100%)	60(66.7%)	30(33.3%)	Total

Comparison of the results of Efficiency of pentoxifylline with verapamil showed reduction in plaque size in patients receiving pentoxifylline was (30%) and in verapamil was (33.3%), respectively. According to fisher exact test there is no significant difference between pentoxifylline and verapamil with verapamil with plaque volume reduction. (P=0.63) And also there is no significant difference between each drugs alone with recipients simultaneously in plaque size reduction (P=0.29) There is no significant difference between pentoxifylline with pentoxifylline and verapamil (P=0.29) (Table 5).

**Table 5:** Comparison of the efficacy of pentoxifylline - Verapamil - Pentoxifylline and Verapamil in plaque size reduction

Total	Plaque size reduction		Treatment with

	Do not have	Have	
30	21(70%)	9(30%)	Pentoxifylline
30	20(66.7%).	10(33.3%).	Verapamil
30	20(66.7%)	10(33.3%)	Pentoxifylline and Verapamil
90	60(66.7%)	30(33.3%)	Total

Efficiency of pentoxifylline in comparison with verapamil showed, improvement of erectile dysfunction in patients receiving pentoxifylline was (46.7%) and in verapamil was (66.7%) and in patients taking pentoxifylline and verapamil was (86.7%). According to Chi-square test there is no significant difference between pentoxifylline and verapamil with reduction in erectile dysfunction. (P=0.05) There is a significant difference between pentoxifylline with pentoxifylline and verapamil (P=0.001) in improving erectile dysfunction. But there is no significant difference between verapamil with pentoxifylline and verapamil in improving erectile dysfunction (P=0.06) (Table 6).

**Table 6:** Comparison of the efficacy of Pentoxifylline - Verapamil - Pentoxifylline and Verapamil in improving erectile dysfunction

Total	Improving erectile dysfunction		Treatment with

	Do not have	Have	
30	16(53.3%)	14(46.7%)	Pentoxifylline
30	10(33.3%)	20(66.7%)	Verapamil
30	4(13.3%)	26(86.7%)	Pentoxifylline and Verapamil
90	30(33.3%)	60(66.7%)	Total

Efficiency of pentoxifylline in comparison with verapamil showed that: of 30 patients who received pentoxifylline 73.3% had pain reduction and 26.7% had no pain reduction and in patients receiving verapamil 76.7% had reduction in pain and 23.3% had no pain relief. According to the Fisher Exact test there is a significant difference between medications in reducing pain. (P=0.05) Also, there is no significant difference between (pentoxifylline and verapamil with verapamil) (P=0.06) and there is the difference between pentoxifylline with verapamil with pentoxifylline (P=0.001) (Table 7).

**Table 7:** Comparison of the efficacy of Pentoxifylline - Verapamil - Pentoxifylline and Verapamil in reducing pain

Total	Pain reduction		Treatment with

	Do not have	Have	
30	8(26.7%)	22(73.3%)	Pentoxifylline
30	7(23.3%)	23(76.7%)	Verapamil
30	6(20%)	24(80%)	Pentoxifylline and Verapamil
90	17(18.9%)	73(81.1%)	Total

Effects observed in patients treated with verapamil were as follows: dizziness in 3 patients, weakness in 5 patients, nausea in 2 patients, and sweating in 7 patients. In pentoxifylline reported side effects were: headache in 3 patients, nausea in 3 patients and vomiting in 4 patients.

## 4. Discussion

Peyronie’s disease with two definitions of fibromatos and penile curvature and erectile dysfunction mostly occur in men aged 40 to 60 (Gonzalez-Cadavid, 2009). The main complaint that makes a visit to the clinic, is of creating fibromatos nodules on the upper surface of the penile, which leads to the curvature and deviation of penile especially during erection, while create no problem with urinary in terms of urine (Pohlers, 2009). But, effective studies have not been conducted yet in connection to efficiency and effectiveness of these two drugs and their combination in the control of symptoms and stop or slow the disease process and improve symptoms. Hence, we investigated the effects of two injectable verapamil and oral pentoxifylline and the combination of these two drugs in the treatment of the amount of pain, the size of the plaque, the amount of circumflexion, erectile dysfunction and the control of progress of the disease and improvement of symptoms of peyronie’s disease. In this study a total of 90 patients with signs and symptoms of the peyronie’s disease diagnosis in the age range 40 to 70 years were studied. In 30 patients who received pentoxifylline, 26.7% curvature reduction, 30% plaque size reduction, 46.7% the recovery of erectile dysfunction and 73.3% pain reduction was reported. Each of these cases were in patients who used verapamil was 36.7%, 33.3%, 66.7% and 76.6%, respectively, in patients, who used verapamil and pentoxifylline, curvature reduction was 36.7%, plaque size reduction was 33.3%, the recovery of erectile dysfunction was 86.7% and pain reduction was 80%. According to the results there is no significant difference between the two drugs used and their function on Peyronie’s disease in terms of curvature reduction, plaque size reduction and pain reduction. However, there was a significant difference between the recovery rate of erectile dysfunction in patients who used verapamil, compared with patients who had been taking pentoxifylline alone with studies conducted by Safarinejad et al. (2009) that analyzed the efficacy and the safety of extended-release pentoxifylline (PTX-SR) in the treatment of patients with basic course chronic Peyronie’s disease. Overall, 36.9% of patients who received PTX-SR had a positive response to treatment. While only 4.5% in the control group stated this. Improvement of erectile dysfunction was reported 34%, plaque size reduction 25%, pain reduction 89.2% and curvature reduction 40% and thus extended-release pentoxifylline has average effect in the treatment of basic course chronic Peyronie’s disease that is consistent with findings of our study. In our study pentoxifylline compared with oral administration of pentoxifylline with verapamil had less effect. In another study by Shindel et al. (2009) had been finally concluded, as regards metabolism and production of collagen and elastin affect regulatory TGF-B1 at TADF cells, the inhibitory effect of pentoxifylline on TGF-B1 causes reduction in the secretion and production of elastin and collagen and these suggestive changes are the beneficial role of pentoxifylline in the treatment of Peyronie’s disease, is consistent. In a study that was conducted by Levine et al. (2002) as a result of intra-plaque injection is an effective treatment in reducing pain and curvature and improve sexual activity and very low rate of complications in the treatment of Peyronie’s disease that the complications observed in our study in patients treated with verapamil are as follows: Dizziness in 3 patients, weakness in 5 patients, and nausea in 2 patients and sweating in 7 patients, in the pentoxifylline reported side effects were: headache in 3 patients, nausea in 8 patients and vomiting in 4 patients was reported. In the comparison of these results with a study conducted by Levine et al. (2002) in terms of reducing pain and improving erectile dysfunction and fewer complications are consistent.

In our study, the softening of plaques was also investigated and the results showed that soft plaque in patients receiving pentoxifylline was 51% and in verapamil was 47% and in recipients simultaneously pentoxifylline and verapamil was 70%. Given that in our study improvement of symptoms of Peyronie’s disease in the group receiving verapamil had no significant difference compared to pentoxifylline, taking pentoxifylline and verapamil simultaneously may improve symptoms of Peyronie’s disease (erectile dysfunction, curvature rate, plaque size, pain) which was significant in terms of improvement of erectile dysfunction and can won more improvement by combination of these two drugs than prescribing pentoxifylline or verapamil.
